# Differential microbiological spectrum and resistance pattern in periprosthetic hip joint infections: a matched-cohort analysis comparing direct anterior versus lateral approach

**DOI:** 10.1186/s12891-022-05037-x

**Published:** 2022-01-19

**Authors:** Alexander Aichmair, Bernhard J. H. Frank, Gabriel Singer, Sebastian Simon, Martin Dominkus, Jochen G. Hofstaetter

**Affiliations:** 1 II. Department of Orthopaedic Surgery, Orthopaedic Hospital Vienna-Speising, Speisinger Straße 109, 1130 Vienna, Austria; 2 Michael Ogon Laboratory for Orthopaedic Research, Orthopaedic Hospital Vienna-Speising, Speisinger Straße 109, 1130 Vienna, Austria; 3 Sigmund Freud University, Freudplatz 3, 1020 Vienna, Austria

**Keywords:** Joint infection, Microorganism, Total hip arthroplasty, Direct anterior approach, Lateral approach

## Abstract

**Background:**

In recent years, total hip arthroplasty via the direct anterior approach (DAA) has become more common. Little is known on the influence of the surgical approach on the microbiological spectrum and resistance pattern in periprosthetic hip joint infections. The aim of the present study was to evaluate the microbiological spectrum and resistance pattern in periprosthetic hip joint infections comparing the direct anterior versus lateral approach in a matched-cohort analysis at a single institution.

**Methods:**

Patients who underwent revision hip arthroplasty due to PJI following primary total hip arthroplasty with culture positive microbiology were analyzed. In all study patients, both the primary surgery and the revisions surgery were performed at the same institution. Only patients in whom primary surgery was performed via a direct anterior or lateral approach were included (*n* = 87). A matched cohort analysis was performed to compare the microbiological spectrum and resistance pattern in PJI following direct anterior (*n* = 36) versus lateral (*n* = 36) primary THA.

**Results:**

We identified both a significantly different microbiological spectrum and resistance pattern in PJI comparing direct anterior versus lateral approach THA. *Cutibacterium avidum* was obtained more frequently in the anterior subgroup (22.2% vs. 2.8%, *p* = 0.028). In the subgroup of infections with *Staphylococcus aureus* (*n* = 12), methicillin resistance was detected in 3/5 cases in the direct anterior group versus 0/7 cases in the lateral group (*p* = 0.045). Overall, *Staphylococcus epidermidis* was the most common causative microorganism in both groups (direct anterior: 36.1%; lateral: 27.8%, *p* = 0.448).

**Conclusion:**

The present study indicates a potential influence of the localization of the skin incision in THA on the microbiological spectrum and resistance pattern in PJI. *Cutibacterium avidum* seemed to be a more common causative microorganism in PJI in patients who underwent direct anterior compared to lateral approach THA.

**Supplementary Information:**

The online version contains supplementary material available at 10.1186/s12891-022-05037-x.

## Background

Periprosthetic joint infections (PJI) are among the most serious complications following total joint arthroplasty, with a reported incidence of up to 2% reported in the literature [1, 2]. Due a rapidly increasing demand for total joint arthroplasties over the next decades, the need for total hip and knee revision surgeries has been reported to rapidly grow within the upcoming decades [3]. In recent years, total hip arthroplasty via the direct anterior approach (DAA) has become more common [4–6]. In THA via the AMIS (*Anterior Minimally Invasive Surgery*) technique, the surgical incision is placed over the tensor fasciae latae muscle and uses the anterior intermuscular and internervous interval [7, 8]. There are antithetic results regarding the risk of PJI following direct anterior THA compared to other approaches [9–13]. While Ilchmann et al. identified no increased risk of infection for the DAA versus the lateral approach [12], Aggarwal et al. reported on a higher rate of PJI in patients who underwent DAA THA compared to other surgical approaches in an analysis of 6086 cases [13]. Identification of the microorganism as well as the specific antimicrobial resistance pattern are paramount for the subsequent treatment in PJI. Prior studies have identified an inferior outcome in the setting of polymicrobial infections [14] and multi-drug resistant microorganisms [15].

A recent study investigated the dermal microbial colonization pattern at the groin, anterior, and lateral thigh. Interestingly, a significantly different colonization pattern was identified, with significant influence of obesity on the dermal colonization at the groin [4]. Despite these findings, little is known whether this approach-specific dermal colonization has an impact on the microbiological spectrum and resistance pattern in periprosthetic hip joint infections comparing the direct anterior versus other approaches. The aim of this study was to perform a single-center matched-cohort analysis on the microbiological spectrum and resistance pattern in periprosthetic hip joint infections comparing the direct anterior versus lateral approach.

## Methods

### Study population

**Fig. 1:**
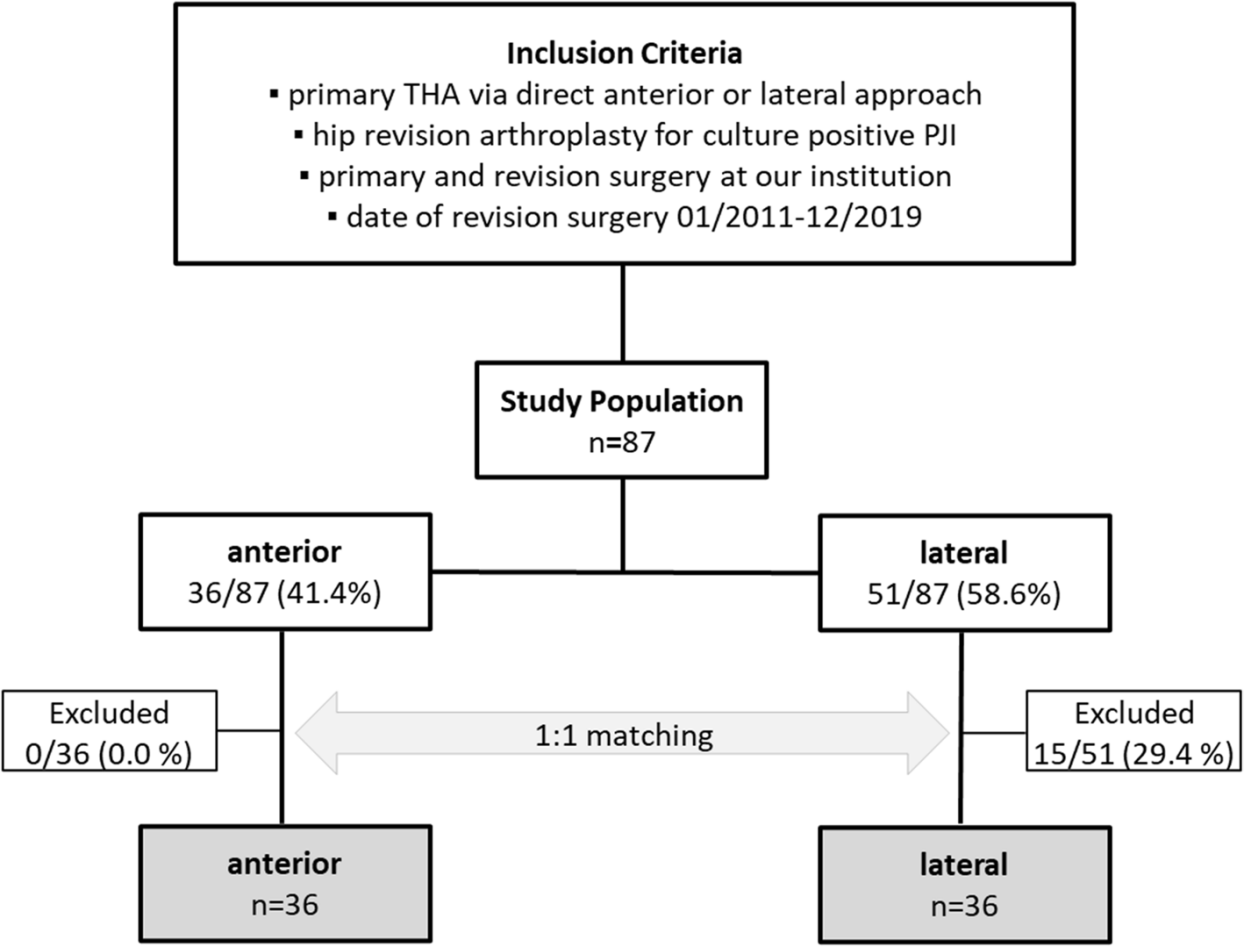
Flow-chart diagram of patient inclusion and exclusion

The present study was approved by the institutional review board (#EK04/2021). We analyzed a total of 1682 patients who underwent either aseptic or septic revision hip arthroplasty at our institution between 01/2011 to 12/2019. Study inclusion criteria were defined as (a) primary THA via the direct anterior or lateral approach, (b) hip revision arthroplasty for culture positive PJI defined by the 2018 Musculoskeletal Infection Society (MSIS) criteria [16], and (c) both primary and revision arthroplasty performed at the same institution. Overall, 87 cases (anterior: 36, lateral: 51) were available for further analysis. (Fig. 1) Data were then collected on age, gender, body mass index (BMI), surgical approach, the interval between primary total hip arthroplasty and revision surgery, the Surgical Site Infection Risk Score (SSIRS) [17], the Charlson Comorbidity Index, in addition to the microbiological analysis and resistance pattern. Both sub-groups were finally matched (1:1) for age, gender, BMI, rate of polymicrobial infections, SSIRS, and the Charlson Comorbidity Index, in a case-by-case manner. The microbiological spectrum and resistance pattern were then compared between the sub-group of patients who had undergone primary THA via the direct anterior (*n* = 36) versus the lateral approach (*n* = 36). Matching was controlled by comparing the matching variables between both sub-groups.

### Surgical revision technique

Based on the type of infection and PJI characteristics, a first stage of two stage procedure, DAIR (debridement, antibiotics and implant retention), a single stage procedure, or a resection arthroplasty (Girdlestone) were performed. During revision surgery, tissue samples were obtained and the explanted device was prepared for sonication analysis. During first stage of two stage procedure, all prosthetic components were removed in addition to synovectomy and debridement. An antibiotic cement spacer was implanted using *Heraeus COPAL® G + C revision cement (Heraeus Medical GmbH, Wertheim, Germany)* with 1 g Gentamicin and 1 g Clindamycin per batch.

### Microbiological analysis

Intraoperative samples were used for the analysis of the microbiological spectrum and the microbiological resistance pattern, as previously published [18]. After covering the explanted devices in a container with sterile saline, it was sonicated in an ultrasound bath for 1 min and vortexed for 30 s. Tissue samples and aliquots (0.1 ml) of the sonication fluid were plated in aerobic and anaerobic sheep blood agar as well as in chocolate agar plates and were inoculated into thioglycollate broth. Cultures were incubated at 35 ± 1 °C for 10 days. A terminal subculture was performed from all thioglycollate broth specimens on blood agar plates and incubated at 35 ± 1 °C for another 4 days. The *BD Phoenix™* automated identification and susceptibility testing system (Becton Dickinson and Company, USA), in addition to standard microbiological techniques, were used for identification and antimicrobial susceptibility testing [18]. Results were further interpreted according to criteria as published by the European Committee on Antimicrobial Susceptibility testing (EUCAST) [19]. The antibiotic treatment was then adapted according to the antibiogram results, our institution’s empiric guidelines, and the institution’s infectious disease specialist.

### Statistical analysis

Categorical variables are reported as frequencies and percentages, and continuous variables as mean ± standard deviation (SD). The Pearson’s chi squared test, or Fisher’s exact test if the expected cell count in any cell was < 5, were used for the comparison of proportions. The Kolmogorov Smirnov test was used to test for a normal distribution. In case of a parametric distribution of continuous data, the Student’s t-test was applied. The Mann-Whitney U test was used for the comparison of continuous variables in case of a non-parametric distribution. A *p*-value of < 0.05 was defined as the level of statistical significance. Data analysis was performed using IBM SPSS Statistics, Version 26.0 (IBM Corp., Armonk, NY).

## Results

### Study population

A total of 87 patients (female: 38, male: 49) met the study’s inclusion criteria. The average age at revision surgery was 67.8 ± 14.1 (range: 21.0–93.3) years. Primary total hip arthroplasty was performed via the direct anterior approach in 36 cases, and via the lateral approach in 51 cases. The average BMI was 29.8 ± 6.2 (range: 16.7–45.7) kg/m^2^, the average SSIRS 8.5 ± 3.7 (range: 3–31), and the average Charlson Comorbidity Index 3.6 ± 2.1 (range: 0–11). In the majority of cases a monomicrobial infection was diagnosed (*n* = 67; 77.0%). Overall, the average number of detected pathogens was 1.3 ± 0.6 (range: 1–4). The five most common microorganisms were *Staphylococcus epidermidis* in 27 cases (31.0%), *Staphylococcus aureus* in 15 cases (17.2%), *Cutibacterium avidum* in 10 cases (11.5%), *Cutibacterium acnes* in 8 cases (9.2%), and *hemolytic Streptococcus (β)* in 6 cases (6.9%).

After matching, two subgroups with a sample size of 36 each (*n* = 72) were available for further analysis (Fig. 1). In the matched cohort, the average age at revision surgery was 68.2 ± 13.6 years, performed after an average of 19.3 ± 34.9 months after primary arthroplasty. The average BMI was 31.3 ± 5.7 kg/m^2^, the SSIRS 9.0 ± 3.7, and the Charlson Comorbidity Index 3.5 ± 1.9. There were no statistically significant differences between both matched cohorts, with regard to age, gender distribution, BMI, rate of polymicrobial infections, SSIRS, and Charlson Comorbidity Index (*p* > 0.05 each).

### Microbiological spectrum

Overall, 426 microbiological cultures were analyzed, with 300 (70.4%) positive cultures. The median number of obtained cultures per patient was 6 (IQR 4–7), with a median of 3 (IQR 2–6) positive cultures. There was no statistically significant difference of obtained cultures (*p* = 0.699) and positive cultures (*p* = 0.565) between the anterior and lateral subgroups. In the majority of cases a monomicrobial infection was diagnosed (*n* = 56, 77.8%). In polymicrobial infections (*n* = 16, 22.2%), the average number of detected pathogens was 2.3 ± 0.7 (range: 2–4). In patients with polymicrobial infections, *Staphylococcus epidermidis* was the most commonly involved microbe (22.2%), half of which were attributable to *Staphylococcus epidermidis* resistant to methicillin (MRSE), followed by *Cutibacterium avidum* (11.1%). Details are listed in Tables 1 and 2.

**Table 1:** Details of the microbiological spectrum of the matched-cohort study population (*n* = 72)

	Overall (***n*** = 72)		Anterior (***n*** = 36)		Lateral (***n*** = 36)		***P***-Value
*Staphylococcus epidermidis*	23	(31.9%)	13	(36.1%)	10	(27.8%)	0.448
*▪ MRSE*	*14/23*	*(60.9%)*	*8/13*	*(61.5%)*	*6/10*	*(60.0%)*	*1.000*
*Staphylococcus aureus*	12	(16.7%)	5	(13.9%)	7	(19.4%)	0.527
*▪ MRSA*	*3/12*	*(25.0%)*	*3/5*	*(60.0%)*	*0/7*	*(0.0%)*	*0.045*
*Cutibacterium avidum*	9	(12.5%)	8	(22.2%)	1	(2.8%)	**0.028**
*Cutibacterium acnes*	6	(8.3%)	2	(5.6%)	4	(11.1%)	0.674
*hemolytic streptococcus (β)*	5	(6.9%)	2	(5.6%)	3	(8.3%)	1.000
*hemolytic streptococcus (γ)*	4	(5.6%)	2	(5.6%)	2	(5.6%)	1.000
*Enterococcus faecalis*	4	(5.6%)	1	(2.8%)	3	(8.3%)	0.614
*Pseudomonas aeruginosa*	3	(4.2%)	2	(5.6%)	1	(2.8%)	1.000
*Staphylococcus capitis*	3	(4.2%)	1	(2.8%)	2	(5.6%)	1.000
*Staphylococcus hominis*	3	(4.2%)	1	(2.8%)	2	(5.6%)	1.000
*Bacillus species*	3	(4.2%)	1	(2.8%)	2	(5.6%)	1.000
*Staphylococcus haemolyticus*	2	(2.8%)	2	(5.6%)	0	(0.0%)	0.493
*Proteus mirabilis*	2	(2.8%)	1	(2.8%)	1	(2.8%)	1.000
*Granulicatella adiacens*	2	(2.8%)	1	(2.8%)	1	(2.8%)	1.000
*Streptococcus viridans*	2	(2.8%)	0	(0.0%)	2	(5.6%)	0.493
*Escherichia coli*	1	(1.4%)	1	(2.8%)	0	(0.0%)	1.000
*Staphylococcus chromogenes*	1	(1.4%)	1	(2.8%)	0	(0.0%)	1.000
*Klebsiella pneumoniae*	1	(1.4%)	1	(2.8%)	0	(0.0%)	1.000
*Corynebacterium tuberculostearicum*	1	(1.4%)	1	(2.8%)	0	(0.0%)	1.000
*Staphylococcus sasscharolyticus*	1	(1.4%)	0	(0.0%)	1	(2.8%)	1.000
*Staphylococcus lugdunensis*	1	(1.4%)	0	(0.0%)	1	(2.8%)	1.000
*Finegoldia magna*	1	(1.4%)	0	(0.0%)	1	(2.8%)	1.000
*Enterobacter cloacae*	1	(1.4%)	0	(0.0%)	1	(2.8%)	1.000
*Candida albicans*	1	(1.4%)	0	(0.0%)	1	(2.8%)	1.000

**Table 2:** Pathogens involved in polymicrobial infections (36 pathogens in 16 polymicrobial infections)

*Staphylococcus epidermidis*	8	(22.2%)
*▪ MRSE*	*4/8*	*(50.0%)*
*Cutibacterium avidum*	4	(11.1%)
*Bacillus species*	3	(8.3%)
*hemolytic streptococcus (β)*	3	(8.3%)
*Cutibacterium acnes*	2	(5.6%)
*Enterococcus faecalis*	2	(5.6%)
*Proteus mirabilis*	2	(5.6%)
*Staphylococcus capitis*	2	(5.6%)
*Candida albicans*	1	(2.8%)
*Enterobacter cloacae*	1	(2.8%)
*Finegoldia magna*	1	(2.8%)
*Granulicatella adiacens*	1	(2.8%)
*Klebsiella pneumoniae*	1	(2.8%)
*Methicillin-resistant Staphylococcus aureus*	1	(2.8%)
*Staphylococcus chromogenes*	1	(2.8%)
*Staphylococcus hominis*	1	(2.8%)
*Staphylococcus lugdunensis*	1	(2.8%)
*Staphylococcus sasscharolyticus*	1	(2.8%)

**Fig. 2:**
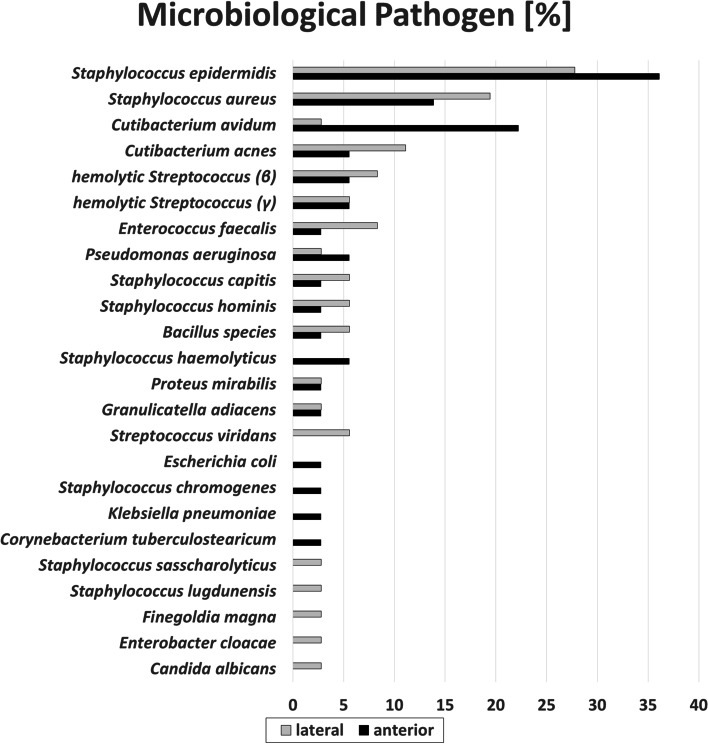
Microbiological spectrum in periprosthetic hip joint infections (matched-cohort, *n* = 72)

Group comparison between patients who underwent direct anterior versus lateral THA showed a significantly different distribution of *Cutibacterium avidum* between both sub-groups. While it was detected in 8 cases (22.2%) in the anterior approach sub-group, it was detected in only 1 case (2.8%) in the lateral approach sub-group (*p* = 0.028). Overall, *Staphylococcus epidermidis* was the most commonly detected pathogen in microbiological analysis, with no statistical difference between patients who underwent anterior (36.1%) versus lateral (27.8%) THA (*p* = 0.448) (Table 1, Fig. 2).

### Surgical treatment of PJI

Overall, a first stage of a two-stage procedure was performed in 35 cases (48.6%), with 13 cases in the anterior subgroup and 22 cases in the lateral subgroup. A single stage procedure was performed in 16 cases (22.2%), with 14 cases in the anterior subgroup and 2 cases in the lateral subgroup. DAIR was performed in 13 cases (18.1%) with 5 cases in the anterior subgroup and 8 cases in the lateral subgroup. A resection arthroplasty (Girdlestone procedure) was performed in 8 cases (11.1%) with 4 cases in each subgroup.

### Resistance pattern

Details of the overall microbiological resistance pattern are listed in Table 3. Patients with a PJI due to *Staphylococcus epidermidis* or *Staphylococcus aureus* showed a resistance to Amoxicillin and Clavulanic acid in 60.9% (14/23) and 25.0% (3/12), respectively. Similar findings were identified for the resistance rate to cephalosporines. In fact, the resistance rate to Cefuroxim was 60.9% (14/23) and 25.0% (3/12) in patients with a PJI due to *Staphylococcus epidermidis* or *Staphylococcus aureus*, respectively. The resistance rate to Clindamycin was 47.8% (11/23) in the cases with *Staphylococcus epidermidis*, and 8.3% (1/12) with *Staphylococcus aureus*. The resistance rate to Cotrimoxazol was low both in cases with *Staphylococcus epidermidis* (9.1%, 2/22) and *Staphylococcus aureus* (0%, 0/10), respectively. Similar resistance rates were observed for Daptomycin and Fosfomycin. There was a very low resistance rate to Linezolid, and Rifampicin. Furthermore, while there was no case of resistance to Teicoplanin in patients with an infection to *Staphylococcus aureus*, the resistance rate was 31.6% (6/19) in *Staphylococcus epidermidis* cases. There was no case of resistance to Vancomycin.

**Table 3:** Details of the microbiological resistance pattern of the matched-cohort study population (*n* = 72)

	***Staphylococcus epidermidis***		***Staphylococcus aureus***		***Cutibacterium avidum***	
Amoxicillin	13/13	(100.0%)	4/4	(100.0%)	0/3	(0.0%)
Amoxicillin + Clavulanic Acid	14/23	(60.9%)	3/12	(25.0%)	0/3	(0.0%)
Ampicillin	13/13	(100.0%)	1/1	(100.0%)	0/3	(0.0%)
Ampicillin + Sulbactam	n/a	n/a	1/1	(100.0%)	0/1	(0.0%)
Cefazolin	14/23	(60.9%)	3/12	(25.0%)	0/3	(0.0%)
Cefepim	13/22	(59.1%)	3/11	(27.3%)	n/a	n/a
Cefotaxim	22/22	(100.0%)	12/12	(100.0%)	6/6	(100.0%)
Cefoxitin	11/18	(61.1%)	3/8	(37.5%)	n/a	n/a
Cefoxitin Screen	8/10	(80.0%)	1/4	(25.0%)	n/a	n/a
Ceftriaxon	13/22	(59.1%)	3/11	(27.3%)	n/a	n/a
Cefuroxim	14/23	(60.9%)	3/12	(25.0%)	n/a	n/a
Cephalexin	n/a	n/a	1/1	(100.0%)	n/a	n/a
Chloramphenicol	1/14	(7.1%)	1/5	(20.0%)	n/a	n/a
Ciprofloxacin	8/21	(38.1%)	1/9	(11.1%)	n/a	n/a
Clarithromycin	15/23	(65.2%)	1/12	(8.3%)	1/4	(25.0%)
Clindamycin	11/23	(47.8%)	1/12	(8.3%)	0/6	(0.0%)
Cotrimoxazol	2/22	(9.1%)	0/10	(0.0%)	n/a	n/a
Daptomycin	2/22	(9.1%)	0/10	(0.0%)	n/a	n/a
Doxycyclin	9/23	(39.1%)	3/12	(25.0%)	n/a	n/a
Erythromycin	9/16	(56.3%)	1/5	(20.0%)	0/1	(0.0%)
Fosfomycin	3/23	(13.0%)	0/12	(0.0%)	n/a	n/a
Fusidic Acid	10/23	(43.5%)	0/12	(0.0%)	n/a	n/a
Gentamicin	5/22	(22.7%)	1/11	(9.1%)	n/a	n/a
Imipenem	n/a	n/a	1/1	(100.0%)	0/3	(0.0%)
Levofloxacin	8/22	(36.4%)	1/11	(9.1%)	n/a	n/a
Linezolid	1/22	(4.5%)	0/11	(0.0%)	n/a	n/a
Mecillinam	22/22	(100.0%)	12/12	(100.0%)	6/6	(100.0%)
Meropenem	n/a	n/a	1/1	(100.0%)	0/6	(0.0%)
Moxifloxacin	6/22	(27.3%)	1/12	(8.3%)	n/a	n/a
Mupirocin	1/4	(25.0%)	0/12	(0.0%)	n/a	n/a
Oxacillin	14/23	(60.9%)	3/12	(25.0%)	n/a	n/a
Penicillin	n/a	n/a	1/1	(100.0%)	0/6	(0.0%)
Penicillin V	n/a	n/a	1/1	(100.0%)	n/a	n/a
Piperacillin	22/22	(100.0%)	12/12	(100.0%)	6/6	(100.0%)
Piperacillin + Tazobactam	n/a	n/a	1/1	(100.0%)	1/3	(33.3%)
Rifampicin	1/22	(4.5%)	2/11	(18.2%)	n/a	n/a
Teicoplanin	6/19	(31.6%)	0/10	(0.0%)	n/a	n/a
Tigecyclin	0/16	(0.0%)	1/5	(20.0%)	n/a	n/a
Vancomycin	0/23	(0.0%)	0/10	(0.0%)	0/6	(0.0%)

In the subgroup of infections with *Staphylococcus aureus* (*n* = 12), Methicillin (Oxacillin) resistance was detected in 3/5 cases in the direct anterior group versus 0/7 cases in the lateral group (*p* = 0.045). Furthermore, statistically significant differences between the anterior approach and lateral approach sub-groups were detected for *Staphylococcus aureus* resistant to Amoxicillin and Clavulanic Acid (*p* = 0.045), Cefazolin (*p* = 0.045), Cefepim (*p* = 0.024), Ceftriaxon (*p* = 0.024), and Cefuroxim (*p* = 0.045) (Table 4).

**Table 4:**
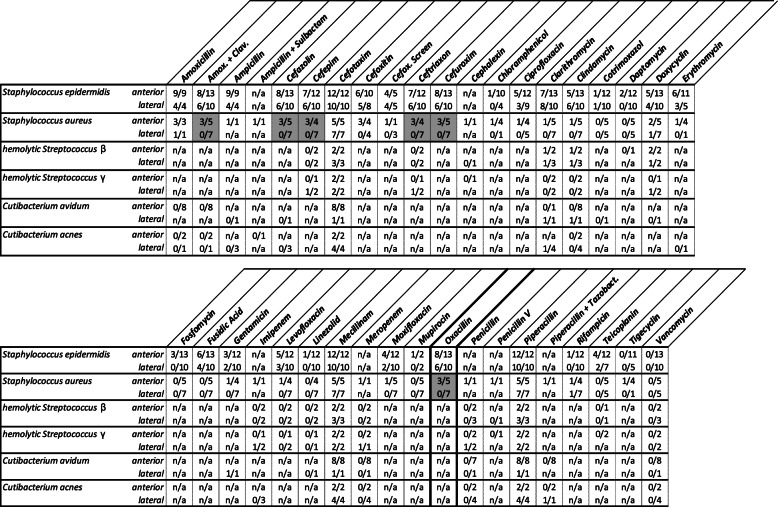
Details of the microbiological resistance pattern of the matched-cohort study population (*n* = 72) for microbiological pathogens with an overall rate of more than 5%. Boxes highlighted in grey indicate a *p*-value < 0.05

## Discussion

This is the first matched-cohort study investigating the microbiological spectrum and resistance pattern in the setting of periprosthetic hip joint infection, with special focus on the location of the surgical incision. We identified significant differences in the microbiological spectrum and resistance pattern in periprosthetic hip joint infections following primary direct anterior versus lateral THA. Subgroup comparison demonstrated a significantly different rate of *Cutibacterium avidum*, a gram-positive anaerobic-aerotolerant rod [20], mainly found in in moist body areas [21]. Interestingly, *Cutibacterium avidum* was the underlying pathogen in the anterior approach THA subgroup in 22.2% compared to 2.8% in the lateral approach THA subgroup (*p* = 0.028). This concurs with the findings by Böni et al. The investigators prospectively evaluated skin scrapings from the groin and thigh in 65 patients undergoing THA. The dermal colonization rate of *Cutibacterium avidum* was reported at 32.3%. Furthermore, the odds ratio per unit BMI increase was calculated at 1.15 (*p* = 0.01). However, due to a relatively small sample size and limited follow-up, no conclusion on the clinical relevance could be drawn [4]. Since the 2018 MSIS criteria [16] were used to define a prosthetic joint infection in our patient population, we believe that *Cutibacterium avidum* should be considered a truly pathogenic bacterium rather than a contaminant.

Similarly, Elkins et al. investigated the *Cutibacterium* colonization of the anterior and lateral thigh. The authors collected dermal punch biopsies in 101 adult patients scheduled for hip or knee surgery. Biopsies were performed after a preoperative skin cleansing protocol (2% chlorhexidine) and a mechanical skin scrub (70% isopropyl alcohol). A total number of 404 cultures were collected at locations approximating a direct anterior and a lateral skin incision, with 22% of patients having a positive culture. The most commonly isolated pathogen was *Cutibacterium* species (65%), with *Cutibacterium acnes* in the majority of cases (87%). Interestingly, 65% of all positive cultures were obtained at the skin area approximating a direct anterior approach. Potentially related to a type II error, the comparison of skin biopsy location (anterior vs. lateral) did not show a statistically significant difference (*p* = 0.076). Interestingly, only in 1 case of a total of 15 cultures positive for *Cutibacterium* sp., *Cutibacterium avidum* was detected [5]. This is in contrast with the results of our study. Overall, in approximately 20.8% of positive cultures, *Cutibacterium* species was detected. When analyzed separately, 12.5% of cultures were positive for *Cutibacterium avidum* as opposed to 8.3% positive for *Cutibacterium acnes* in the present study.

Based on these findings we agree with the conclusion of other investigators [4, 5] that current skin disinfection protocols should be evaluated for their efficacy to reduce *Cutibacterium* species skin colonization of the anterior thigh and groin area. Interestingly, Maurer et al. reported a preoperative groin colonization with *Cutibacterium avidum* in 20% of patients undergoing THA. Routine skin antisepsis was reported to be ineffective in 40 and 20% after the first and third antisepsis, respectively. The skin antisepsis protocol consisted of Betaseptic® solution (Mundipharma, Limburg, Germany), which is povidone-iodine, 2-propanol and ethanol. Kodan® (Schülke, Norderstedt, Germany) was utilized in patients with an allergy against iodine, which is 2-Propanol, 1-Propanol, Biphenyl-2-ol, hydrogen peroxide and purified water. Due to a limited sample size, follow-up studies are warranted [22].

Overall, *Staphylococcus epidermidis* was the most commonly detected pathogen in the setting of PJI following primary THA, with however no significant difference between the anterior and lateral approach THA subgroups. Another main finding of the present study is the fact that *Staphylococcus aureus* resistant to Oxacillin (i.e. MRSA subspecies) was only obtained in the anterior approach subgroup. In the subgroup of infections with *Staphylococcus aureus* (*n* = 12), Methicillin (Oxacillin) resistance was detected in 3/5 cases in the direct anterior group versus 0/7 cases in the lateral group (*p* = 0.045). This concurs with the findings by Yang et al., investigating body site colonization with MRSA in patients with acute *Staphylococcus aureus* skin and soft tissue infection. An overall of 37% of patients presented with a colonization, with inguinal MRSA colonization reported in 11%. Interestingly, non-nasal colonization was 25% among patients with community-associated MRSA, as opposed to 6% with community-associated *Staphylococcus aureus* susceptible to methicillin (i.e. MSSA subspecies) [23]. In immunocompromised patients, the rate of MRSA colonization was reported to range between 13 and 15%. In 21% colonization was detected at the groin area only, and in 38% at both the groin and nasal area [24].

In cases with a beta-lactam allergy, Clindamycin is frequently administered for preoperative surgical prophylaxis [25]. However, according to our findings, there seems to be a high resistance rate to Clindamycin in cases with *Staphylococcus epidermidis*. Follow-up studies with a larger sample size, especially with regard to resistance pattern analysis, are warranted to identify more potent antibiotics for preoperative prophylaxis in the context of allergies. Resistance rate analysis further identified Cotrimoxazol, Daptomycin, Fosfomycin, and Linezolid as antibiotics with relatively low resistance rates in cases with *Staphylococcus epidermidis* and *Staphylococcus aureus*. In fact, Cotrimoxazol has been demonstrated to be an effective antibiotic, which can be used as a salvage therapy in bone and joint infections, even in the setting of infections with gram-negative bacilli, polymicrobial infection, and orthopedic device infections [26]. Our data underline the important role of Cotrimoxazol (Sulfamethoxazole-Trimethoprim, SXT) in the treatment of aggressive periprosthetic joint infections with low resistance rates. Despite the low rate of resistance to Daptomycin and Fosfomycin in cases with *Staphylococcus epidermidis* or *Staphylococcus aureus*, potential downsides need to be considered. First, Daptomycin requires intravenous administration and may have nephrotoxic side effects. Second, Fosfomycin may only be administered in combination with other antibiotics. No cases of resistance to Vancomycin were identified.

Based on our clinical experience, we believe that PJI cases with *Cutibacterium avidum* as the underlying pathogen, should be treated according to the current treatment regimen recommendations. Surgical treatment protocols include debridement and implant retention, one-stage, or two-stage revision surgery, depending on the time course of onset of infection, pathogenicity of the underlying microbe, and patient-related factors including soft-tissue or bone quality, among others. In cases with difficult-to-treat microorganisms, fistula, or prior failed revision surgeries, a two-stage exchange should be considered rather than one-stage exchange [27]. With regard to antibiotic therapy, recommended antibiotics with a low rate of resistance include β-lactams, fluoroquinolones, macrolides, and rifampin. Resistance to erythromycin and clindamycin has been described [20].

The present study represents the largest of its kind with regard to sample size and study design, to the best of our knowledge. Nevertheless, limitations need to be considered when interpreting the reported findings. Especially with regard to the resistance pattern, the sample sizes were too small in order to perform a multivariate analysis. However, a matched cohort analysis was alternatively performed to account for potential confounding. In fact, both sub-groups were matched for a variety of variables to focus on the influence of the surgical approach on PJI.

## Conclusion

*Cutibacterium avidum* seemed to be a more common causative microorganism for PJI in patients who underwent THA via the direct anterior compared to the lateral approach. We therefore believe that current skin disinfection protocols need to be evaluated in terms of their efficacy to reduce surgical site contamination by *Cutibacterium* species. A higher rate of resistance to Methicillin (Oxacillin) in PJI with *Staphylococcus aureus* was detected in the direct anterior versus the lateral group, which however warrants further research.

## Supplementary Information

**Additional file 1.**

## Data Availability

All data generated or analysed during this study are included in this published article and its supplementary information files.
